# The skin microbiome: the overlooked axis in modern dermatology

**DOI:** 10.1097/MS9.0000000000005009

**Published:** 2026-04-21

**Authors:** Rania Saad Ibrahim Abdelghany Elbeshbishy, Adnan Khatak

**Affiliations:** a Department of Internal Medicine, Faculty of Medicine, Ain Shams University, Cairo, Egypt; b Department of Internal Medicine, Nangarhar University, Jalalabad, Afghanistan

**Keywords:** acne vulgaris, antimicrobial stewardship, atopic dermatitis, cutaneous dysbiosis, microbiome-directed therapy, skin microbiome

## Abstract

The skin microbiome plays a critical role in maintaining cutaneous barrier integrity, modulating immune responses, and influencing the expression of dermatologic disease, yet its integration into clinical practice remains limited. Microbial alterations have been described in conditions such as atopic dermatitis, acne vulgaris, psoriasis, and chronic wounds; however, uncertainty regarding causality, interindividual variability, and lack of methodological standardization have hindered clinical translation. Current diagnostic frameworks rarely incorporate microbial metrics, while antimicrobial therapies remain central to management, often without consistent consideration of their ecological impact. Emerging microbiome-directed strategies, including topical probiotics, bacteriophage therapy, and microbiome-preserving approaches, show early promise but lack robust clinical validation. Advancing the role of the skin microbiome in dermatology will require standardized research methodologies, integration of multi-omics approaches, and well-designed clinical trials with clinically meaningful outcomes. This editorial highlights the need for a balanced and evidence-based framework that incorporates microbial perspectives into dermatology without overstating current evidence, advocating for a gradual integration that complements established immunologic and barrier-focused paradigms.

## Introduction

Over recent decades, dermatology has achieved significant advances in understanding the structural, immunologic, and genetic foundations of skin disease; however, one essential dimension of cutaneous health, the resident microbial ecosystem, has received comparatively limited attention in both research and clinical settings. The skin microbiome, composed of diverse bacterial, fungal, viral, and arthropod communities, contributes to barrier stability, immune modulation, and inflammatory regulation, yet its relevance is frequently overshadowed by dominant frameworks that emphasize barrier dysfunction or immune dysregulation[1].

This relative underrepresentation persists despite a growing body of evidence demonstrating distinct microbial signatures across conditions such as atopic dermatitis, acne, psoriasis, and chronic wounds, where shifts in community composition often correlate with disease onset or severity. Translation of these insights into clinical practice remains limited; as diagnostic pathways rarely incorporate microbial metrics, antimicrobial therapies continue to dominate management strategies, and existing guidelines seldom address stewardship or the long-term preservation of microbial balance[2].

This editorial places the skin microbiome back into focus as an important, though historically overlooked, axis in dermatology, with the aim of clarifying where current evidence provides meaningful insight into disease mechanisms. By examining both the scientific landscape and the structural factors that have constrained clinical integration, this discussion advocates for a balanced and methodologically rigorous incorporation of microbiome science into contemporary dermatologic thought, in a manner that complements rather than displacing established paradigms.

## Editorial

### The cutaneous microbiome

The skin hosts a diverse and site-specific microbial ecosystem composed of bacteria, fungi, viruses, and microscopic arthropods that collectively contribute to cutaneous equilibrium. A well-established body of literature shows that microbial communities vary predictably according to anatomical locations, influenced by factors such as moisture, pH, temperature, and sebaceous activity. Sebaceous regions tend to favor *Cutibacterium* species, while moist or intertriginous areas commonly support *Staphylococcus* and *Corynebacterium* populations[3]. These organisms play important roles in limiting pathogenic colonization through competitive interactions, reinforcing barrier function, and shaping innate immune responses at the skin surface. Advances in sequencing and metagenomic techniques have expanded understanding of these relationships, with experimental work demonstrating that commensal species such as *Staphylococcus epidermidis* can modulate antimicrobial peptide production and promote immunologic balance. These findings support the view that the microbiome actively participates in maintaining cutaneous homeostasis rather than existing as a passive collection of microorganisms[1].

Despite this progress, many aspects of skin microbiome function remain incompletely defined. Interindividual variation is substantial and appears to be shaped by genetic factors, environmental exposures, hygiene practices, and topical treatments, which complicates the identification of consistent microbial patterns. Determining whether microbial shifts represent causal contributors to disease or secondary responses to inflammation and barrier disruption is an ongoing challenge, and discrepancies in sampling techniques, sequencing platforms, and analytic methods further limit comparability across studies. Current evidence underscores the microbiome’s importance in cutaneous biology, yet also highlights the need for cautious, methodologically rigorous interpretation to ensure that emerging insights are integrated into dermatologic understanding in a balanced and evidence-based manner^[^2,3^]^.

### Why the skin microbiome remains underrepresented in clinical dermatology

Although interest in the skin microbiome has grown rapidly, its impact on daily dermatologic practice remains modest, largely because clinical reasoning in dermatology has been historically shaped by immunologic and barrier-focused models that have accumulated strong evidence over several decades. These established frameworks provide clear diagnostic pathways and therapeutic strategies, while microbial ecology often remains a secondary consideration in disorders that are not primarily infectious in nature. Practical barriers also limit integration, since sequencing technologies lack standardization, require specialized interpretation, and remain inaccessible for routine clinical use. Microbiome assessments do not yet offer validated diagnostic thresholds, which further reduces their relevance in guiding clinical decisions[3].

Therapeutic conventions reinforce this limited role, as many common dermatoses are treated with antimicrobial agents that offer symptomatic benefit but may alter commensal diversity. Stewardship principles, although increasingly recognized in other medical disciplines, are not consistently emphasized in dermatology, and long-term ecological impacts are rarely incorporated into management plans. In addition, though associations between dysbiosis and skin disease continue to emerge, many remain correlational, and uncertainties surrounding causalities make it difficult to translate findings into practice. Together, these historical, methodological, and evidence-related factors explain why the skin microbiome, despite its scientific relevance, remains underrepresented in clinical dermatology^[^1,3^]^.

### Microbiome and disease associations

Across several common dermatoses, research has identified microbial patterns that accompany changes in disease activity; however, the strength and consistency of these associations vary considerably. Atopic dermatitis is one of the most extensively studied conditions, with multiple investigations reporting increased colonization by *Staphylococcus aureus* during flares and reduced microbial diversity compared to healthy skin, yet uncertainty remains regarding whether these microbial shifts are causal or secondary to barrier disruption and immune alteration^[^3,4^]^.

In acne vulgaris, research involving *Cutibacterium acnes* has shown that disease severity is linked more to specific phylotypes with differing inflammatory potential than to overall bacterial abundance; however, heterogeneity across studies limits clear clinical interpretation. Psoriasis presents a more complex picture, as reports of altered *Malassezia* representation or changes in bacterial taxa are inconsistent across cohorts, likely reflecting differences in sampling sites, sequencing platforms, and analytic methods^[^1,4^]^.

Chronic wound research offers more actionable insights, particularly regarding the role of biofilms in persistent inflammation and delayed tissue repair, although the organisms involved vary with host and environmental factors. Taken together, current evidence indicates that microbial signatures hold relevance across multiple dermatologic diseases; however, many associations remain preliminary, context dependent, or limited by methodological variability. A careful and evidence-based approach is required to distinguish robust patterns from findings that still require stronger validation.

### Therapeutic implications

Growing interest in the cutaneous microbiome has encouraged exploration of therapies that aim to modulate microbial communities rather than eliminate them; however, the evidence supporting these approaches remains early and variable. Studies of topical probiotics and postbiotics suggest that selected microbial strains or their metabolic products may influence barrier function, inflammation, or colonization resistance; however, most findings are derived from small clinical studies or mechanistic research with limited generalizability.

Bacteriophage therapy has emerged as a potential method for targeting specific pathogens such as *Staphylococcus aureus* while preserving commensal diversity, yet questions regarding delivery, durability, and safety keep it largely investigational[3]. Microbial transplantation, involving the transfer of presumed beneficial commensals, shows preliminary promise but faces substantial challenges related to donor selection, formulation, regulation, and reproducibility.

More conventional approaches, including reducing dependence on broad-spectrum antimicrobials and selecting treatments that minimize disruption of commensal populations, have been proposed as microbiome-conscious strategies, yet they are not widely incorporated into clinical guidelines^[^4^]^. Across these modalities, gaps in evidence persist, particularly regarding long-term follow-up, standardized outcomes, and replication across diverse populations. As a result, microbiome-directed therapies should be regarded as hypothesis generating rather than practice altering. Progress will depend on rigorously designed clinical trials and clearer mechanistic understanding to determine which strategies hold meaningful potential for future dermatologic care.

### Integrating microbiome science into dermatologic practice

Integrating microbiome science into dermatologic practice requires a practical and measured approach that acknowledges both the potential value of microbial insights and the limitations of current evidence. Antimicrobial stewardship represents an immediate and feasible entry point, as many common dermatoses are managed with repeated or broad-spectrum antibiotics that may improve symptoms but disrupt commensal diversity[2].

Clinicians may also consider how topical agents such as antiseptics, emollients, and corticosteroids influence resident microbial communities, particularly in chronic conditions that require long-term management. Although routine microbiome sequencing is not yet practical for most clinical settings, future selective evaluation of microbial patterns in recurrent or refractory cases may provide useful insight into persistent colonization or biofilm formation that contributes to treatment resistance, and ongoing advances may allow these assessments to serve as adjunctive tools[5].

A broader shift in perspective is also necessary, recognizing the microbiome as an integral component of the cutaneous ecosystem and encouraging therapeutic decisions that account for ecological effects without overstating their immediate clinical significance. Choosing interventions that minimize unnecessary disruption of commensal populations or pairing standard treatments with strategies that support barrier health reflects a gradual and evidence-informed incorporation of microbiome science into clinical care.

### The way forward

Advancing the role of the skin microbiome in dermatology requires research efforts that are methodologically consistent and clinically relevant. Standardized approaches to sampling, sequencing, and data interpretation are essential for improving comparability across studies, and clearer definitions of dysbiosis may help distinguish meaningful microbial changes from normal variation[5].

Longitudinal cohort studies and multi-omics techniques can further clarify functional relationships between microbial communities and host tissues, while carefully validated artificial intelligence tools may assist in identifying complex and clinically relevant patterns. Rigorous clinical trials with standardized outcomes and adequate follow-up remain essential to determine the translational value of microbiome-based interventions. Through coordinated interdisciplinary efforts, a more integrated and evidence-based dermatologic framework can gradually emerge.

## Conclusion

The expanding body of research on the skin microbiome underscores its relevance to cutaneous health and disease; however, its integration into dermatologic practice must remain guided by careful interpretation of the available evidence. Microbial communities influence barrier function, immune activity, and disease expression; however, the extent and consistency of these effects vary across conditions, emphasizing the need for rigorous and standardized investigation. As the field continues to evolve, dermatology has an opportunity to incorporate microbial perspectives in a manner that complements established frameworks grounded in immunology and barrier science. A balanced and evidence-based approach that acknowledges both the potential and the current limitations of microbiome research will be essential. Thoughtful integration of microbial insights can strengthen dermatological understanding while maintaining scientific precision and clinical responsibility.

## Data Availability

No datasets were generated or analyzed during this study; therefore, data sharing is not applicable.
